# Tuning the Structure–Property Relationships in Binary and Ternary Blends of PLA/PBAT/PHBH

**DOI:** 10.3390/polym16121699

**Published:** 2024-06-14

**Authors:** Mohammadreza Nofar, Reza Salehiyan, Massimiliano Barletta

**Affiliations:** 1 Sustainable & Green Plastics Laboratory, Metallurgical & Materials Engineering Department, Faculty of Chemical and Metallurgical Engineering, Istanbul Technical University, Istanbul 34469, Turkey; 2 School of Computing, Engineering and the Built Environment, Edinburgh Napier University, Edinburgh EH10 5DT, UK; r.salehiyan@napier.ac.uk; 3 Dipartimento di Ingegneria, Università degli Studi Roma Tre, Via Vito Volterra 62, 00146 Roma, Italy; massimiliano.barletta@uniroma3.it

**Keywords:** polylactide, poly(3-hydroxybutyrate-co-3-hydroxyhexanoate), polybutylene adipate terephthalate, binary blends, ternary blends

## Abstract

While the brittle polylactide (PLA) has a high durability among bioplastics, poly(3-hydroxybutyrate-co-3-hydroxyhexanoate) (PHBH) with certain ductility exhibits facile compostability. The addition of polybutylene adipate terephthalate (PBAT) may also be used to improve the ductility and toughness of brittle bioplastics. Binary and ternary blends of PLA/PBAT/PHBH based on either PLA or PHBH as the matrix have been manufactured using a twin-screw extruder. The melt rheological, mechanical, and morphological properties of the processed samples were examined. Binary blends of PLA/PHBH show superior strength, with the PLA75/PHBH25 blend exhibiting a tensile strength of 35.2 ± 3.0 MPa, which may be attributed to miscible-like morphology. In contrast, blends of PLA with PBAT demonstrate low strength, with the PLA50/PBAT50 blend exhibits a tensile strength of 9.5 ± 2.0 MPa due to the presence of large droplets in the matrix. PBAT-containing blends exhibit lower impact strengths compared to PHBH-containing blends. For instance, a PLA75/PBAT25 blend displays an impact strength of 1.76 ± 0.1 kJ/m^2^, whereas the PHBH75/PBAT25 blend displays an impact strength of 2.61 ± 0.3 kJ/m^2^, which may be attributed to uniformly dispersed PBAT droplets.

## 1. Introduction

The vast usage of various petroleum-based and non-degradable plastics such as polystyrene (PS), polyethylene terephthalate (PET), polyethylene (PE), and polypropylene (PP) has generated a serious threat for nature and the Earth [1]. Bioplastics may be placed in one of three major groups, either biobased, biodegradable, or biobased and biodegradable [2]. Biodegradability and compostability can be considered to be an essential feature for plastics in commodity applications. At the end of their lifetime, the majority of these are deposited in landfills. Biodegradability and compostability of bioplastics is dependent on environment [3,4]. For instance, while polyhydroxybutyrate (PHB) and its copolymers, starch, cellulose, and cellulose acetate are fully compostable in marine, fresh water, soil, home composting, industrial compositing, anaerobic digestion, and landfills, polylactide (PLA), which is known as the most well-developed commercial bioplastic, can mainly only be composted in industrial composting and anaerobic digestion environments. Some other bioplastics such as PBAT and PBSA can be composted in soil, home composting, and industrial compositing environments [5,6,7,8,9]. These differences are primarily due to chemical structure differences and hence various reactivity differences in various environments. The biodegradability and compostability of bioplastics is highly dependent on glass transition temperature (T_g_) and the degree of crystallinity [10]. Degradability occurs most readily around and beyond the T_g_ due to molecular flexibility, which permits greater exposure to degrading bacteria. Similarly, the more compacted molecular structure in crystallites may impede the degradation due to the reduced molecular mobility and diffusivity. This means that the biodegradability of PLA, for instance, may be facilitated by low crystallinity and at temperatures close to and beyond T_g_ (~60 °C) [10]. Therefore, the chemical structure of the bioplastic, the portion of crystallinity and environmental conditions such as temperature, humidity, etc. can dramatically influence degradability versus durability for various bioplastics. The durability of bioplastics can also be enhanced by introducing branched structures. The number of reactive groups may be reduced by the chain extension, while increased molecular entanglements can hinder molecular mobility [11]. In this context, the composites based on various bioplastics could also reflect different degradability versus durability depending on the incorporated filler type and content [12].

Blending various bioplastics to generate a new type of material with desired thermal, physical, mechanical, and viscoelastic properties can also lead to tailoring their biodegradability or durability in various environments [12,13,14]. While for some commodity applications longer service time is required, for some other cases long lifetime is not required. Therefore, blending various bioplastics with different lifetimes and service temperatures with respect to degradability and with controlled blending ratios can be an effective approach, generating a new series of bioplastic materials with desired biodegradability or durability. The properties of blends can be optimized by controlling the ratio of each polymer and adjusting the processing conditions during the blend preparation. It should be well-noted that the morphology of the blend, including the size and distribution of the dispersed phases, and their interfacial interactions dramatically influence the properties as well as the biodegradability mechanisms of the final products. The careful design and control of the preparation process to obtain the desired properties of the blend is essential. In this context, the blending of brittle and durable PLA with highly ductile and tough polybutylene adipate terephthalate (PBAT) or polybutylene succinate-co-adipate (PBSA) can determine the mechanical properties of the final blend [15,16,17,18]. The biodegradability of PLA can also be influenced by the addition of PBAT, while PHB copolymers such as poly(3-hydroxybutyrate-co-3-hydroxyvalerate) (PHBV) or poly(3-hydroxybutyrate-co-3-hydroxyhexanoate) (PHBH) degrade rapidly. Degradation may also occur under most environmental conditions [19,20]. Hence, blending of these materials with durable bioplastics such as PLA could be a promising route to tailoring degradability [21]. The use of PBAT could also affect the degree of degradation of the final blend. However, the influence on ductility and toughness and overall processability of the blend is more crucial [6,20,21,22]. The rheological and mechanical performance and morphologies of binary and ternary blends of PLA/PBAT/PHBH have been explored. For various compositions, melt viscoelastic, mechanical, and thermal performance have been calculated.

## 2. Experimental

### 2.1. Materials 

**Figure 1:**
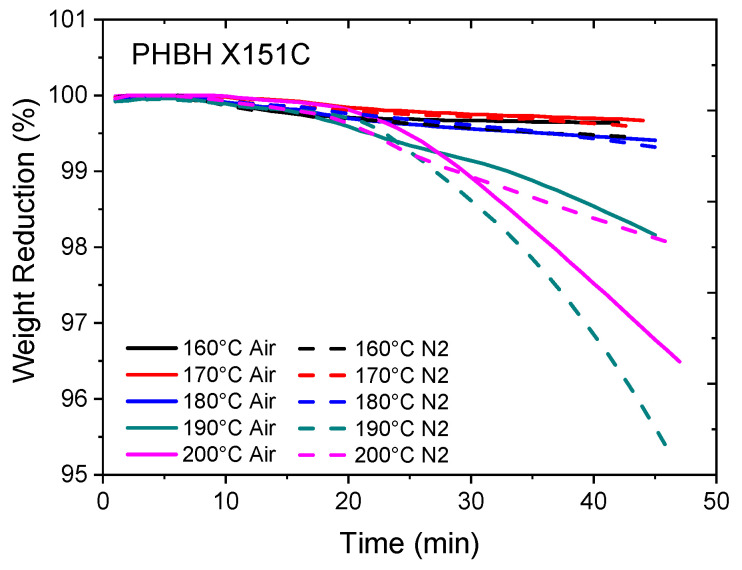
Thermal stability analysis of PHBH X151C under air and nitrogen using isothermal TGA.

A commercial linear and amorphous PLA (Ingeo 10361D) with a D-lactide content of 12 mol% was supplied by NatureWorks LLC, USA. The PBAT used is Ecoflex^®^ F Blend C1200 from BASF, a grade suitable for blown film or cast film processing. PHBH X151C with a melt flow index of (165 °C, 5 kg) 3 g/10 min and HH-content of 10 mol% was supplied by KANEKA. The isothermal thermogravimetric analysis (TGA) of PHBH X151C at various temperatures and under air or nitrogen is presented in Figure 1. This figure illustrates the sensitivity of PHBH to the thermal degradation, even in a nitrogen environment. 

**Figure 2:**
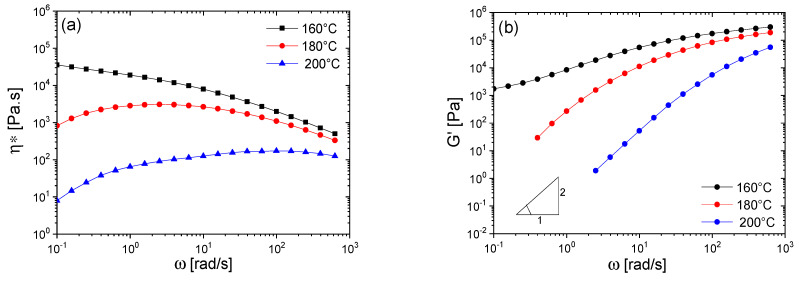
Complex viscosity (**a**) and storage modulus (**b**) of PHBH X151C at various testing temperatures.

Polyesters are known to be prone to thermo-oxidative degradation, where their viscosity can increase at higher temperatures and in the presence of air due to trans-esterification and branching in the early stages. This explains why the degradation rate at 200 °C in air is somewhat slower. This behavior is observed in the viscosity results shown in Figure 2, where an increasing trend in viscosity against frequency is evident for PHBH at 180 and 200 °C.

The small amplitude oscillatory shear (SAOS) rheological analysis of the noted PHBH at 160, 180, and 200 °C is shown in Figure 2, which further demonstrates the thermal degradation sensitivity of this PHA material. As observed, PHBH undergoes significant thermal degradation beyond 160 °C. Amorphous PLA was selected to enable melt processing of the blends at temperatures as low as 150 °C in order to minimize the degradation of PHBH, which is extremely sensitive to thermal degradation.

### 2.2. Blend Preparation 

Binary and ternary blends were prepared with either PLA as the matrix and PHBH and/or PBAT as minor phases, or PHBH as the matrix and PLA and/or PBAT as the minor phases. These blends were formulated at weight ratios (wt%) of 75/25, 75/12.5/12.5, 50/50, and 50/25/25 using a twin-screw extruder (TSE) (Gülnar Makina Ltd., Melikgazi, Turkey) with a co-rotating screw diameter of 16 mm and an L/D ratio of 30. The screw speed was set at 100 rpm, and the temperature profile was adjusted at 50-130-150-150-150-150 °C (from hopper to the die). Prior to processing, all formulations were weighed, dry mixed, and dried in a vacuum oven at 50 °C overnight. Rheological, tensile, and impact testing samples were prepared using a compression molding machine at 150 °C for 5 min under 1.5-ton pressure.

To predict the morphology of the ternary blend systems, the thermodynamic Harkins spreading equation was employed. In ternary blends, different morphologies can be obtained according to their intermolecular interactions. In context with intermolecular interactions, the spreading coefficient was defined by Harkins equation as follows [23,24]:

(1) $$\lambda_{AB}=\gamma_{BC}-\gamma_{AC}-\gamma_{AB},$$

where the interfacial tensions between polymers are represented by γ and each polymer pair is represented by the indices. $\gamma_{AB}$ can be theoretically predicted using the geometric and harmonic mean equations [25,26] (Equations (2) and (3)).

(2) $$\gamma_{AB}=\gamma_{A}+\gamma_{B}-2\left(\sqrt{\gamma_{A}^{d}\gamma_{B}^{d}}+\sqrt{\gamma_{A}^{p}\gamma_{B}^{p}}\right)$$

(3) $$\gamma_{AB}=\gamma_{A}+\gamma_{B}-4\left[\frac{\gamma_{A}^{d}\gamma_{B}^{d}}{\gamma_{A}^{d}{+\gamma }_{B}^{d}}+\frac{\gamma_{A}^{p}\gamma_{B}^{p}}{\gamma_{A}^{p}+\gamma_{B}^{p}}\right]$$

$\gamma_{A}$ and $\gamma_{B}$ are the surface energies of polymers A and B, respectively, where d and p represent polar and dispersive contributions of the surface energy of polymers. In order to calculate the interfacial tension between the components at the processing conditions, the surface energies of the components at processing temperatures are needed to calculate the interfacial energies according to the harmonic and geometric mean equations (Equations (2) and (3)). The values of surface energies for PLA, PBAT, and PHBH were obtained from previous reports [27]. The estimated values of surface energies at 25 and 150 °C and their corresponding interfacial tensions are shown in Table 1 and Table 2, respectively. Thus, employing Harkins equation (Equation (1)), the spreading coefficients of the ternary systems were calculated and are shown in Table 3.

According to Equation (1), $\lambda_{AB}$ describes the tendency of component A to engulf component B in a component C matrix. A complete wetting morphology can be generated when component A completely spreads over B and separates B and C. A positive value of $\lambda_{AB}$ indicates the existence of such morphology. On the other hand, partial wetting morphologies can occur when all three phases meet along a line, with droplets of one phase located at the interface between the other two phases [28]. In such cases, the sign of a spreading coefficient will be negative. The spreading coefficient can also become zero, indicating a transient morphology that can change from partial wetting to complete wetting. It should be noted that the geometric mean equation provides more accurate predictions of interfacial tensions of both low and high surface energy materials [29]. Thermodynamically, using the geometric mean equation approach, complete wetting morphologies could be obtained where PLA could separate PBAT and PHBH droplets and completely wet PBAT. From the values shown in Table 3, it can be seen that geometric mean values for spreading coefficients of PHBH/PLA are positive in most instances, while the spreading coefficient values of PLA/PBAT and PBAT/PHBH are all negative. This indicates that droplets of PBAT and PHBH are dispersed within the PLA matrix. However, all these are only thermodynamic predictions, and the actual morphology needs to be examined using scanning electron microscopy (SEM). Many other parameters, such as characteristics of each phase, processing type and conditions, and blend compositions, can also play important roles.

### 2.3. Rheological Analysis

Small amplitude oscillatory shear (SAOS) rheological analysis of the processed samples was conducted using an MCR-301 rotational rheometer (Anton Paar, Graz, Austria) equipped with parallel-plate geometry (25 mm diameter) with a gap of 1 mm. The SAOS rheological experiments were conducted at 150 °C under nitrogen atmosphere. Frequency sweep experiments were conducted at strain amplitudes of 0.01, which were within their linear viscoelastic regions.

### 2.4. Mechanical Properties Analysis

The tensile properties of the samples were investigated using a Zwick/Roell with a 5 kN load cell at 5 mm/min rate following ASTM D638. The samples were dog-bone shaped with length of 25 mm, width of 4 mm, and thickness of 2 mm. A minimum of three specimens were tested for each sample. The impact strength of notched samples was determined using the Zwick/Roell impact device following ASTM D256. The samples were rectangular with a length of 25 mm, width of 4 mm, and thickness of 2 mm, and a minimum of five specimens were tested.

## 3. Results and Discussion

**Figure 3:**
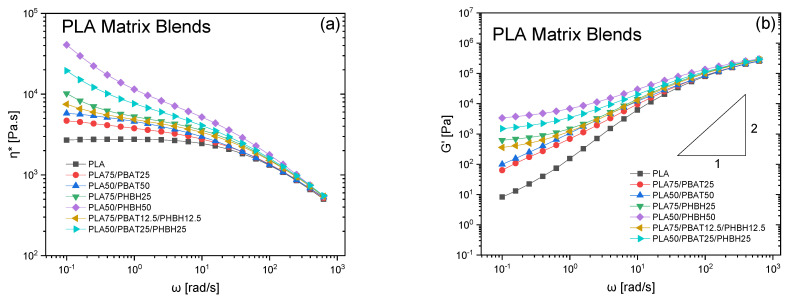

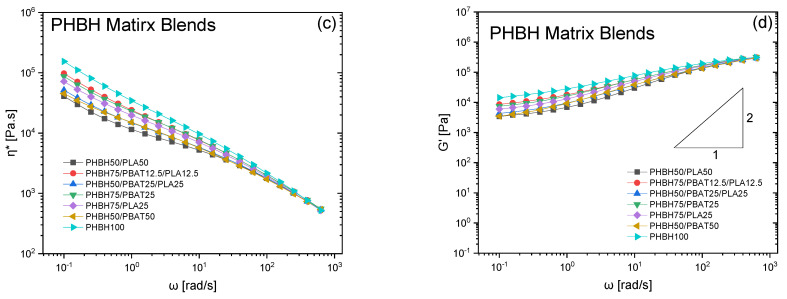
(**a**,**c**) Complex viscosity and (**b**,**d**) elastic moduli (G’) of the blends with (**a**,**b**) PLA or (**c**,**d**) PHBH matrices under small amplitude oscillatory shear (SAOS) tests at nitrogen atmosphere, 150 °C, and strain amplitude of 0.01.

Figure 3 represents the rheological properties of both binary and ternary blends, with either PLA (Figure 3a,b) or PHBH (Figure 3c,d) serving as the matrix. Upon examining the results, it becomes evident that a wide range of responses can be achieved depending on the compositions. Several governing parameters define the final properties, including composition ratios and viscosity ratios of the polymer pairs. These parameters significantly influence the resulting morphologies, which in turn dictate the rheological responses. Notably, when PLA serves as the matrix, the highest elastic modulus and viscosity are obtained when blended with 50% PHBH. It is crucial to discuss the results in relation to the type of minor phase dispersed into the matrix. As illustrated in Figure 3c,d, PHBH exhibits significantly higher viscosity $\eta^{*}$ and G’(ω) than neat PLA. Therefore, blends with PHBH dispersed in a PLA matrix are expected to exhibit higher values than those blends with PBAT droplets. Let us discuss the results based on the viscosity ratios now. Considering the situation where a phase with relatively higher viscosity at 150 °C is dispersed into a lower viscosity matrix, similar to what is shown in Figure 3a,b, there is a significant difference in the rheological responses of the blends with the same dispersed phase content (PHBH or PBAT). This is due to the different inherent viscosities of the two polymers, which determine the viscosity ratios and, in turn, the morphologies. When PBAT is introduced, the viscosities and elastic moduli G’(ω) of the blends increase without changing the slopes in the terminal regions (low frequencies), indicating that the enhancements are primarily due to the more elastic nature of PBAT imparted into the PLA matrix. This results in higher viscosities and elastic moduli, but the fundamental behavior in the terminal region remains unchanged, reflecting the influence of the PBAT’s viscoelastic properties on the blend. However, blends with PHBH dispersed into PLA display a strong plateau behavior (non-terminal) at lower frequencies (Figure 3b). This, in return, leads to more complex morphologies where the viscosity ratio is larger due to the much higher viscosity of the minor phase ($\eta_{d}=$ PHBH here) compared to the matrix ($\eta_{m}=$ PLA) K=ηdηm. This is because, at viscosity ratios above a certain critical threshold, it becomes unlikely for minor phase droplets to undergo breakup [30]. Consequently, the two phases, such as PLA and PHBH, intertwine without experiencing fragmentation into smaller droplets. Therefore, this complex morphology leads to a longer relaxation process at low-frequency regions, causing a more pronounced plateau behavior. Longer relaxation processes corresponding to the plateau modulus at low-frequency regions in blends are associated with the relaxation of the dispersed phase. In this case, PHBH, which has much higher viscosity, cannot relax within the measuring timeframe, resulting in a very pronounced plateau modulus.

Conversely, when PBAT is introduced at the same composition, the rheological properties exhibit notable differences, lacking the plateau region observed in PLA/PHBH blends. In contrast, this reveals a classical droplet-matrix morphology, which typically indicates a lower elastic modulus compared to the more complex pseudo co-continuous structures found in PLA/PHBH blends.

In contrast to blends with PLA as the matrix, the rheological responses of blends with PHBH as their matrices are primarily dictated by the PHBH’s inherent high viscosities. In other words, the magnitude of changes in blends where PHBH is the matrix is comparatively less remarkable. That is due to the smaller viscosity ratios in these blends where PHBH is the matrix (Figure 3c,d). In these blends, the non-terminal plateau modulus extends to higher frequencies, with the overall slopes of the curves being much smaller compared to those in the plateau regions of Figure 3b where PLA was the matrix. This is primarily due to the larger interfaces imposed by the morphologies, which prolong the relaxation of the minor phase in these blends.

Furthermore, the ternary blends exhibited inferior behavior compared to their respective binary blends. For example, the values of PLA50/PBAT25/PHBH25 were lower than those of PLA50/PHBH25. This may be attributed to the morphology evolution, where the addition of PBAT and reduction in PHBH content altered the viscosity ratios, leading to the formation of droplet morphology. Interestingly, the results indicate that all blends converge to a single point at very high frequencies, analogous to higher shear rates, suggesting that pronounced differences may emerge at conditions with smaller displacement rates, such as those at very low frequency ranges. However, discussions regarding the relationship between morphologies and rheological responses are inherently limited, as the observed impact-fractured surface morphologies were obtained at room temperature, while rheological responses are influenced by rather smaller deformations (0.01 strain amplitude) of molten blends. It is worth noting that the blends are not compatibilized; hence, the possibility of coalescence during temperature stabilization and frequency tests cannot be discounted. In our previous work we observed comparable trends when examining the behavior of (70/30) PLA/PVDF over time, noting a significant decrease in the elastic modulus G’(t) within the initial minutes of testing. This reduction, notably more pronounced compared to neat PLA, highlights the impact of coalescence in un-compatibilized blends on diminishing rheological properties [31]. Therefore, it can be concluded that viscosity ratios and blend ratios are key factors in this context. Given that PHBH demonstrates the highest viscosity values, blends containing high PHBH ratios exhibit higher moduli and viscosity values.

**Figure 4:**
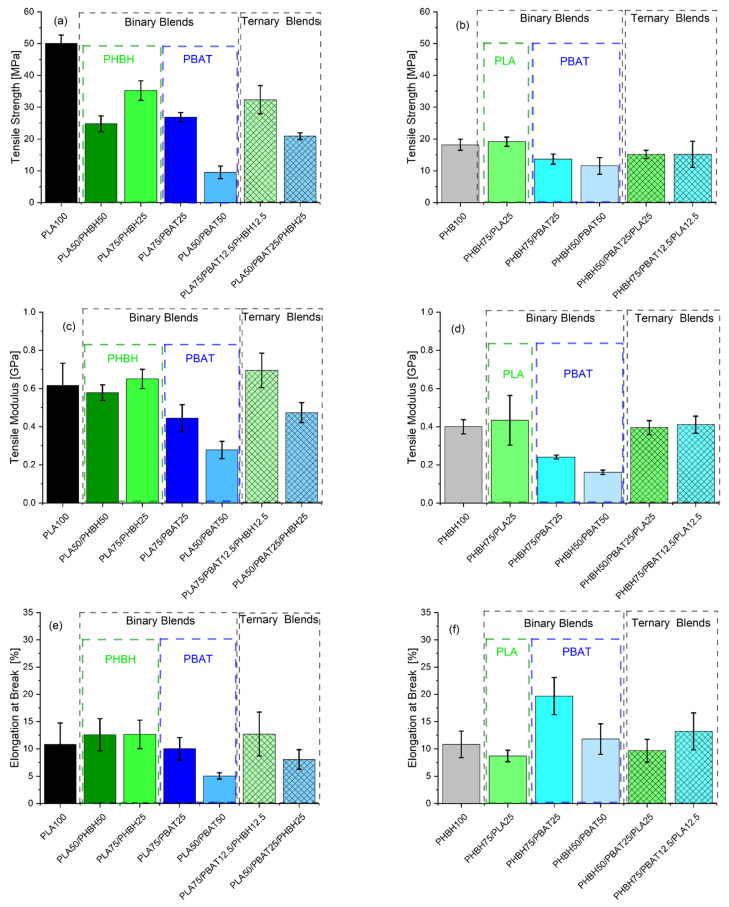
(**a**,**b**) Tensile strength, (**c**,**d**) tensile moduli, and (**e**,**f**) elongation at breaks of binary and ternary blends based on either (**a**,**c**,**e**) PLA or (**b**,**d**,**f**) PHBH. The inset names in green or blue indicate the type of minor phase in the binary blends.

**Figure 5:**
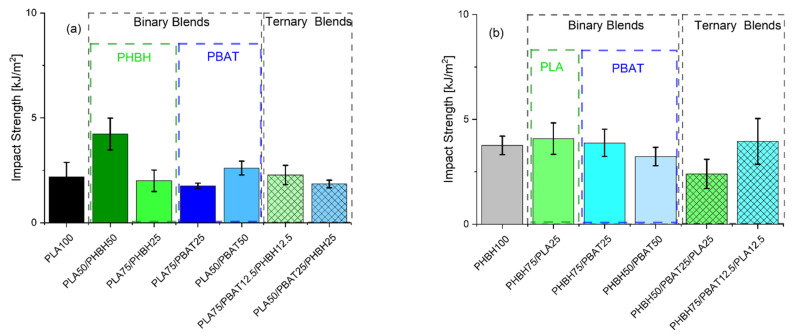
Notched impact strength of the binary and ternary blends based on either (**a**) PLA or (**b**) PHBH. The inset names in green or blue indicate the type of minor phase in the binary blends.

**Figure 6:**
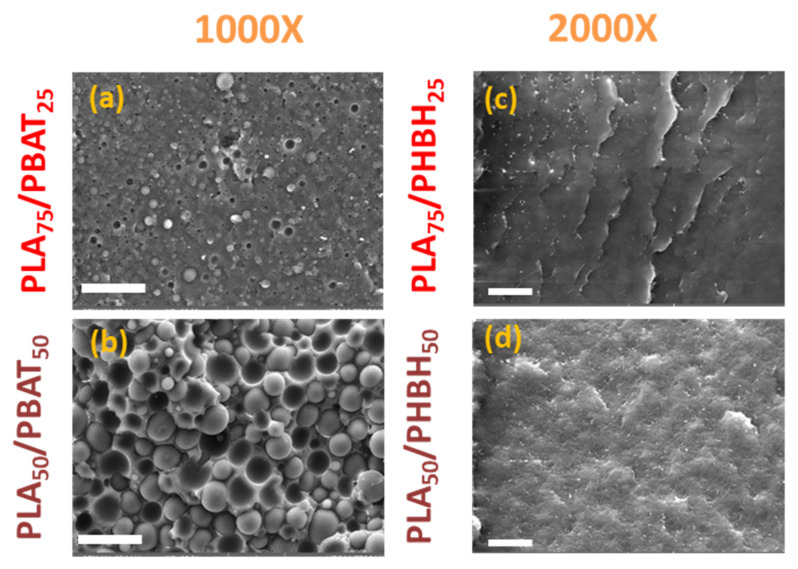
SEM images of the binary blends with PLA as the matrix. The scale bars in (**a**,**b**) PLA/PBAT and PLA/PHBH blends (**c**,**d**) are 50 and 20 µm, respectively.

**Figure 7:**
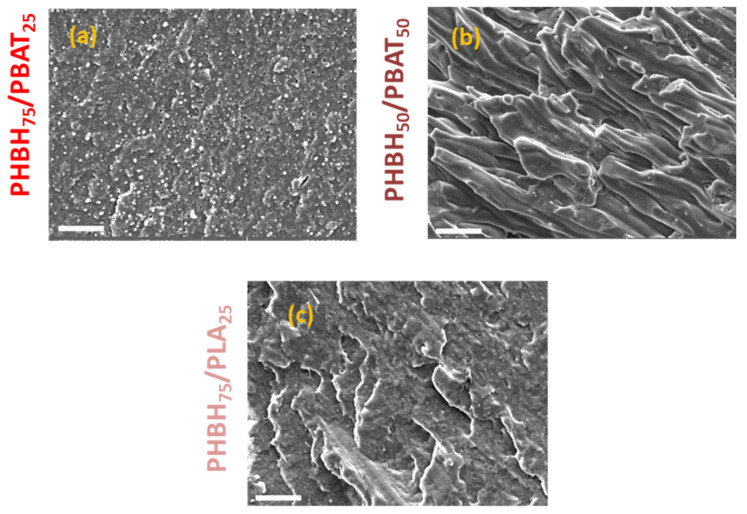
SEM images of the binary blends with PHBH as the matrix. (**a**) PHBH75/PBAT25, (**b**) PHBH50/PBAT50, and (**c**) PHBH75/PLA25. The scale bars and magnifications are 20 µm and 2000 µm, respectively.

**Figure 8:**
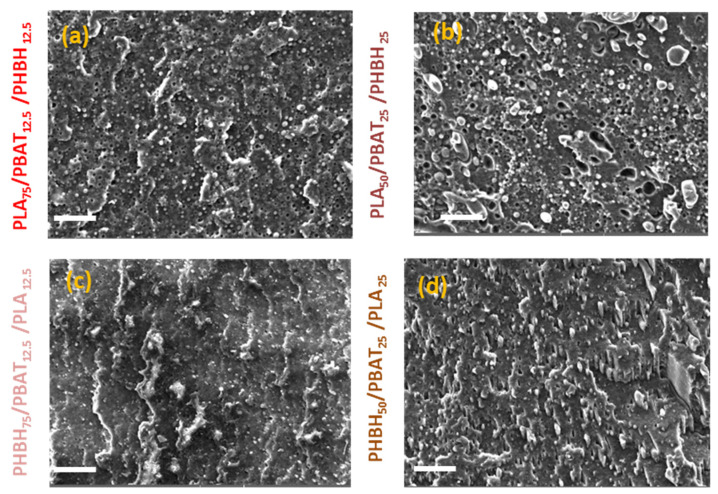
SEM images of the ternary PLA-based (**a**) PLA75/PBAT12.5/PHBH12.5, (**b**) PLA50/PBAT25/PHBH25 and PHBH-based (**c**) PHBH75/PBAT12.5/PLA12.5 and (**d**) PHBH50/PBAT25/PLA25 blends. The Scale bars and magnifications are 20 µm and 2000 respectively.

Figure 4 and Figure 5, respectively, show the tensile and impact properties of binary and ternary blends. Unlike the rheological properties, tensile properties are a reflection of deformation at solid state, where crystal structures of the polymers are preserved. Therefore, blends can behave in a different manner than what they did under molten state. The first glance of Figure 4 reveals a broad range of tensile strength and elongation at breaks. To facilitate the discussion, it would be easier to classify the results based on the binary and ternary blends. In binary blends, as shown in Figure 4a, PLA75/PHBH25 exhibits the largest tensile strength (35.2 ± 3.0 MPa), somewhat intermediate between the neat PLA and PHBH, following the rule of mixture. As expected, addition of further PHBH (50 wt.%) with lower strength 18 ± 1.7 MPa drops the strength of the blends in PLA50/PHBH50 to 24.7 ± 2.5 MPa (≈30% decrease). Overall, the higher strength of PLA/PHBH blends may be ascribed to the co-continuous-like morphology of this blend (Figure 6c,d) to its counterpart droplet morphology PLA/PBAT blends (Figure 6a,b). Considering the morphology of PLA50/PBAT50 (Figure 6b), it is understandable why this specific blend exhibits the lowest strength (9.5 ± 2.0) and elongation at break. Large droplets of PBAT dispersed in the PLA matrix are clearly visible. Comparing it to PLA75/PBAT25 (Figure 6a), where droplets are noticeably smaller, results in higher tensile strength in this case (26.8 ± 1.5). This indicates a reduction of 65.5% in tensile strength and 50% in elongation at break, with a further increase in PBAT content from 25 wt.% to 50 wt.%. Moving forward to ternary blends, it is seen that blends with 75 wt.% PLA and PHBH show the highest elongation at breaks among all ternary blends (12.7 ± 4.00) and (13.3 ± 3.3), respectively. One might assume that elongation at break values should mirror the impact toughness of the blends. However, this is more complicated in immiscible blends, where the interface plays an important role in transferring the imposed load (gradual in tensile tests or sudden in impact tests) to the bulk of the blend being analyzed. Here, SEM images of the impact-fractured surfaces (Figure 6, Figure 7 and Figure 8) can indeed be useful to further understand the behavior of such blends under impact test. Figure 5a,b reveals the impact strength of binary and ternary blends with PLA (Figure 5a) and PHBH (Figure 5b) as their matrices respectively.

As expected, binary blends with PBAT as the minor phase (droplets) show a lower impact strength (Figure 5). Interestingly, PLA50/PBAT50 blends with larger PBAT droplets (Figure 6b) show a greater impact strength 2.61 ± 0.3 kJ/m^2^ compared to the blend with smaller PBAT droplets (Figure 6a), PLA75/PBAT25 (1.76 ± 0.1 kJ/m^2^). This is due to the inherently tougher PBAT phase and the populated honeycomb structure of this blend (Figure 6b). It should be noted that, during the blending process, immiscible polymer pairs undergo intensive shearing, causing the dispersed droplets to experience a series of consecutive breakup and coalescence events. This can lead to the formation of enlarged droplets in immiscible blends because the two polymers are not emulsified. Consequently, two approaching droplets, in this case PBAT, can collide and merge into larger droplets. The chances of frequent collisions are higher when the PBAT content is greater, as seen in the case of PLA50/PBAT50 (Figure 6b).

Moreover, blends with PHBH as the matrix exhibit relatively high impact strengths. This may be attributed to their morphologies, which reveal a ductile fracture pattern (Figure 7). In the case of PHBH75/PBAT25, the PBAT droplets are observed to be extremely small and uniformly dispersed, contributing to the enhanced properties (Figure 7a).

Ternary blends, on the other hand, all exhibit more complicated morphologies. However, the blend having 75 wt.% PHBH (Figure 8c) mirrors the elongation at break results. The lowest impact strength (Figure 5b) belongs to the blends showing droplet-like morphology (Figure 8b) (1.85 ± 0.1 kJ/m^2^).

## 4. Conclusions

This study delved into the fabrication and characterization of binary and ternary blends featuring PLA, PHBH, and PBAT at various weight ratios using a twin-screw extruder. Through comprehensive rheological, tensile, and impact testing, an understanding of the interplay between blend compositions and resulting material properties emerged.

The rheological responses of the blends were found to be tied to the morphological structures dictated by the composition ratios and viscosity ratios of the polymer pairs. Blends with PLA as the matrix exhibited distinct behaviors depending on the type of minor phase dispersed, with PLA/PHBH blends showcasing a complex, non-terminal plateau behavior at lower frequencies attributed to their co-continuous morphology. Conversely, blends with PBAT exhibited classical droplet-matrix morphologies and lower elastic moduli. Similarly, blends with PHBH as the matrix were primarily influenced by PHBH’s high viscosities, resulting in less remarkable changes compared to PLA-based blends.

Tensile properties reflected the solid-state deformation characteristics, with blends demonstrating a wide range of strength and elongation at breaks. Binary blends with PLA/PHBH exhibited higher strength attributed to their co-continuous morphology, whereas blends with PBAT showed reduced strength due to the presence of large droplets. Ternary blends, although exhibiting more complex morphologies, followed similar trends, with blends containing higher PHBH ratios mirroring higher elongation at breaks.

Impact testing revealed relationships between morphology and toughness, with PBAT-containing blends exhibiting lower impact strengths, while blends with PHBH as the matrix displayed relatively high toughness, especially when PBAT droplets were uniformly dispersed.

This comprehensive investigation asserts the importance of blend compositions and morphological structures in determining the mechanical properties of these polymer blends, offering valuable insights for their potential applications in various fields.

## Figures and Tables

**Table 1:** Surface energies of different PLAs, PHBHs, and PBAT at 25, 150, and 200 °C. [27].

Components	Surface Energy at (mJ/m²) 25 °C			Surface Energy at (mJ/m²) 150 °C		
	γ	γd	γp	γ	γd	γp
PLA [1]	39.4	33.6	5.8	31.04286	26.45714	4.514286
PLA [1]	50	37	13	39.42857	29.14286	10.21429
PLA [1]	51.1	34.9	16.1	40.24286	27.75714	12.67143
PLA [1]	53.5	37	16.5	42.14286	29.14286	13
PLA [1]	40.7	32.5	8.2	32.05714	25.57143	6.414286
PLA [1]	53.5	26.8	24.6	42.14286	22.15714	19.38571
PLA [1]	43.5	39.6	3.9	34.28571	31.17143	3.042857
PBAT [1]	38.4	32.1	6.3	30.25714	25.31429	4.942857
PHBH [2]	42.2	33.7	8.5	33.24895	26.55189	6.697064

**Table 2:** Calculated interfacial tensions between PLA, PHBH, and PBAT at 150 °C according to harmonic and geometric mean equations from the values taken from Table 1.

γ (PLA/PBAT) (mJ/m²)		γ(PLA/PHBH) (mJ/m²)		γ(PHBH/PBAT) (mJ/m²)	
Harmonic	Geometric	Harmonic	Geometric	Harmonic	Geometric
0.12	0.09	0.49	0.28	0.29	0.15
2.17	1.15	0.92	0.50		
3.32	1.65	1.68	0.77		
3.89	2.04	2.14	1.09		
0.26	017	0.09	0.08		
9.38	5.45	7.17	4.09		
1.13	0.60	1.81	0.97		

**Table 3:** Calculated values of spreading coefficients according to Equation (1).

PLA/PBAT/PHBH					
λ PLA/PBAT (mJ/m²)		λ PBAT/PHBH (mJ/m²)		λ PHBH/PLA (mJ/m²)	
Harmonic	Geometric	Harmonic	Geometric	Harmonic	Geometric
−0.32	−0.23	0.09	0.04	−0.67	−0.34
−2.80	−1.51	−1.54	−0.80	0.96	0.50
−4.71	−2.28	−1.93	−1.03	1.34	0.74
−5.73	−2.99	−2.04	−1.10	1.46	0.80
−0.07	−0.10	−0.46	−0.23	−0.13	−0.06
−16.26	−9.40	−2.51	−1.51	1.92	1.22
−2.65	−1.43	0.39	0.22	−0.98	−0.51

## Data Availability

The original contributions presented in the study are included in the article, further inquiries can be directed to the corresponding author.
